# Evaluating implementation effectiveness and sustainability of a maternity waiting homes intervention to improve access to safe delivery in rural Zambia: a mixed-methods protocol

**DOI:** 10.1186/s12913-020-4989-x

**Published:** 2020-03-12

**Authors:** Elizabeth G. Henry, Thandiwe Ngoma, Jeanette L. Kaiser, Rachel M. Fong, Taryn Vian, Davidson H. Hamer, Peter C. Rockers, Godfrey Biemba, Nancy A. Scott

**Affiliations:** 1 Department of Global Health and Population, Harvard T.H. Chan School of Public Health, 665 Huntington Avenue, Boston, MA 02115 USA; 2 Right to Care-Zambia, Lusaka, Zambia; 3 Department of Global Health, Boston University School of Public Health, 801 Massachusetts Avenue, Crosstown 3rd Floor, Boston, MA 02118 USA; 4 School of Nursing and Health Professions, University of San Francisco, 2130 Fulton Street, San Francisco, CA 94117 USA; 5 Section of Infectious Diseases, Department of Medicine, Boston Medical Center, Boston, USA; 6 National Health Research Authority, Pediatric Centre of Excellence, Lusaka, Zambia

**Keywords:** Implementation science, Process evaluation, Maternity waiting homes, Zambia, Maternal health, Mixed methods, Fidelity

## Abstract

**Background:**

In low-income countries such as Zambia, where maternal mortality rates are persistently high, maternity waiting homes (MWHs) represent one potential strategy to improve access to safe delivery, especially for women living in remote areas. The Maternity Homes Access in Zambia project (MAHMAZ) is evaluating the impact of a MWH model on women’s access to safe delivery in rural Zambia. There is a growing need to understand not only the effectiveness of interventions but also the effectiveness of their implementation in order to appropriately interpret outcomes. There is little evidence to guide effective implementation of MWH for both immediate uptake and to promote sustainability in this context. This protocol describes a study that aims to investigate the effectiveness of the implementation of MAHMAZ by not only documenting fidelity but also identifying factors that influence implementation success and affect longer-term sustainability.

**Methods:**

This study will use mixed methods to evaluate the implementation effectiveness and sustainability of the MAHMAZ intervention. In our study, “implementation effectiveness” means to expand beyond measuring fidelity to the MWH model and includes assessing both the adoption and uptake of the model and identifying those factors that facilitate or inhibit uptake. Sustainability is defined as the routine implementation of an intervention after external support has ended. Quantitative methods include extracting data from existing records at the MWHs and health facilities to analyze patterns of utilization, and conducting a routine health facility assessment to determine facility-level factors that may influence MWH implementation and woman-level outcomes. We will also conduct an experience survey with MWH users and apply a checklist to assess fidelity to the MWH model. Qualitative methods include in-depth interviews and focus group discussions with MWH users, community members and other stakeholders. Qualitative data will be analyzed using an integrated framework drawing constructs from the Consolidated Framework for Implementation Research and the Conceptual Framework for Sustainability.

**Discussion:**

The findings from this evaluation will be shared with policymakers formulating policy affecting the implementation of MWH and may be used as evidence for programmatic decisions by the government and supporting agencies in deciding to take this model to scale.

**Trial registration:**

NCT02620436, Registered 3 December 2015, Prospectively registered (clinicaltrials.gov; for the overarching quasi-experimental impact study).

## Background

The World Health Organization (WHO) strongly recommends that pregnant women have access to skilled care at every birth as a fundamental strategy to reduce maternal mortality and include support for non-medical interventions that can improve access to safe and supportive maternal delivery environments [1]. In low-income countries such as Zambia, where rates of maternal death are persistently high [2], the use of maternity waiting homes (MWHs) ahead of delivery represents one strategy to increase access to skilled care at birth and reduce maternal mortality, especially for remote women who face distance challenges to reaching health facilities. MWHs are residential accommodations located near health facilities. The intention is for women to use these spaces in advance of their due date in order to improve the likelihood that, in the event of an obstetric emergency, they can be given appropriate care on time, either at the affiliated health center or through referral to a higher-level health facility. There are no specific admission criteria for women to use MWHs, though some facilities encourage women to stay in MWH if they have either maternal or antenatal risk factors for complications. MWH are typically used by women in later stages of pregnancy (1–2 weeks before their estimated delivery date) with priority given to those who live farthest from the facilities.

There is mixed evidence on the effectiveness of MWHs in improving maternal or neonatal health outcomes [3–7]. One systematic review in 2012 identified three quasi-experimental studies that demonstrated reductions in stillbirths from the use of MWH [4], and for one of these studies, conducted in Zimbabwe, the reduction was statistically significant [7]. One study from Liberia showed that communities with MWH had significantly lower rates of maternal death than communities without a MWH, but the post-only with comparison study design limits attribution to the MWH [6]. Another systematic review also from 2012 found that no randomized controlled trials (RCT) of MWH interventions exist, and determined that there was insufficient evidence to determine the impact of MWH on either maternal or neonatal outcomes [3]. In Zambia, one study suggests that use of MWHs may reduce the risk of perinatal mortality, though the evidence itself is relatively weak [8]. In response to requests for additional evidence on the effectiveness of MWHs in the Zambian context, the Maternity Homes Alliance (MHA) was established in 2015. This partnership coordinates efforts of the government of the Republic of Zambia (GRZ), Boston University and Right to Care Zambia (BU/RTC), formerly the Zambian Center for Applied Health Research and Development, and Africare and the University of Michigan (Africare/UM). Funded by Merck Sharp and Dohme (MSD) for Mothers, the Bill & Melinda Gates Foundation, and The ELMA Foundation, the MHA hypothesizes that offering women access to safe, comfortable, and community-acceptable MWHs will bring women closer to quality, facility-based delivery and postpartum care, ultimately improving health outcomes.

As part of the MHA initiative, formative research was conducted to inform the development of community appropriate MWHs [9]. Based on this work, the Core Maternity Waiting Home Model (Core Model) was developed to guide implementation of MWHs, ensuring attention to key components identified as important to local communities and stakeholders (Table 1). Core intervention components consist of: (1) infrastructure, equipment and supplies; (2) governance & management structures and financial management systems; and (3) linkages with the health facility and services offered. A fourth component to support the Core Model includes a financial sustainability strategy with multiple revenue streams.

**Table 1:** MAHMAZ Intervention Components and Features

MWH Intervention Components	Purpose	Features
1. Infrastructure, Equipment & Supplies	Implementation of a high quality, structurally sound MWH that is designed and furnished to be comfortable, safe, and meet community standards of acceptability as defined by a formative evaluation.	• Lighting (lanterns) • Lockable doors, windows • Cooking area and supplies • Bathing and laundry areas • Latrines • Beds, bedding, & bed nets • Staff room (for storage, office, etc.) • Space for postnatal women/newborns to stay • Functional equivalence: concrete floors, no leaky roofs and water
2. Governance, Management & Finances	Formation of governance and management structures made up of local community members to oversee the long-term vision and daily operations of the MWH, adhering to established policies and standard operating procedures, and ensuring compliance with financial management procedures.	• Formalized management and governance structures with government and facility representation • Clear definition of ownership (land, material assets, income generated) • Revenue and asset management • Standard operating procedures (SOPs) for clear roles and responsibilities • Mechanism for community/women’s feedback • Intake, registration, and monitoring procedures • Eligibility: prioritize women living > 10 km from health facility, available for postnatal stays
3. Linkages with Health Facilities & Services	Establishment of close linkages between the MWH and the health facility, including educational classes for waiting women.	• Adjacent to BEmONC within 2 h of a CEmONC facility • Daily end-of-day check-ins by facility staff • ANC and PNC visits conducted at health facility • Emergency transport system identified • Family planning/post-partum family planning education • Breastfeeding and infant and young child feeding education • Education on newborn danger signs, well-baby care • Education on antenatal and postpartum period • Entertainment, recreational activities
4. Financial Sustainability Strategy using Income-Generating Activities (IGA)	Creation of a financial sustainability model to fund the operations and maintenance of the MWH, with revenue derived from various sources, including community donations, health facility donations, and the creation of income generating activities (IGAs). The IGAs are managed by the MWH governance committees and function as social enterprises, generating revenue to operate and maintain the MWH.	• Selection and implementation of one of three IGAs by each site: ▪ A hammermill to grind maize, the local staple crop ▪ An agro-dealership to sell agricultural inputs as well as dry goods ▪ A goat rearing business, to raise and sell goats for meat • Financial management and financial literacy training and in-service mentorship for governance committee members

BU/RTC and Africare/UM are responsible for implementing and evaluating the project as a whole. While the Core Model guides the development of MWHs under the MHA, BU/RTC and Africare/UM were each responsible for developing separate implementation plans that resulted in slightly different strategies for construction, governance and management, linkages with the health facility, and financial sustainability. The MAHMAZ project, implemented by BU/RTC, is operating MWHs in Nyimba District of Eastern Province and Kalomo, Choma and Pemba Districts of Southern Province. While BU/RTC and Africare/UM have a set of harmonized indicators for comparison, MHA partner sites under Africare/UM in Mansa and Chembe Districts in Luapula Province and Lundazi District in Eastern Provinces are not included in this protocol.

The MHA partners are evaluating the impact of the Core Model using a quasi-experimental, controlled before-and-after design in 40 clusters, comparing health centers that receive the Core Model with health centers that continue to operate under standard of care for waiting women [10]. The subset of 20 MAHMAZ sites are randomized to either the intervention arm, implementing the Core Model, or the control arm, implementing the standard of care, resulting in a cluster-randomized controlled trial (cRCT) in the MAHMAZ sites. When implementing the standard of care, health facilities do one of several things. Some facilities may not allow women to wait at the facility for delivery requiring them to come only when they are in labor, some allow them to stay within the facility wards when there is space, and others allow them to use a previously community-constructed MWH (not adhering to the Core Model). The impact evaluation is testing the impact of the intervention on key population-based indicators among women of reproductive age, with the primary outcome being facility delivery. The details of the cRCT, as part of the overall quasi-experimental study, are described elsewhere [10].

There is a growing need to understand not only the effectiveness of interventions but also the effectiveness of the implementation of the interventions in order to appropriately interpret program outcomes [11]. MWHs have been implemented in countries within sub-Saharan Africa with varying degrees of success in terms of utilization [3]. Factors that affect both MWH utilization and women’s satisfaction with their stay include overall MWH quality, cost of staying at the shelter (food, transport), lack of privacy, lack of respect from health staff, limited access to services, being away from family, and safety concerns [12–14]. Research from the community level in Zambia has highlighted barriers to MWH utilization, including lower status of women in decision-making, prevailing norms, and financial challenges [15, 16]. There are also issues with the lack of basic social and healthcare needs in existing MWHs [16]. At the same time, a recent study from Zambia indicates that increased MWH quality may improve the rate of facility delivery among women in the community, regardless of the health facility’s capacity for handling obstetric emergencies [17].

This protocol (v3.1, Oct 26, 2016) complements the impact evaluation [10] (trial registration number NCT02620436) and will investigate the effectiveness of the implementation of MAHMAZ by not only documenting fidelity but also identifying factors that influence implementation success and affect longer-term sustainability. Though some of the tools and methods are also conducted in the Africare sites for a pooled analysis, this protocol addresses implementation effectiveness in the MAHMAZ sites only. In our study, “implementation effectiveness” means to expand beyond measuring fidelity to the Core Model and includes assessing both the adoption and uptake of the model as well as identifying factors that facilitate or inhibit uptake. We defined “sustainability” for this study as the routine implementation of the intervention and continued benefit to women after external support has ended, as well as the processes that affect this [18]. An assessment of intervention effectiveness, using a theory-driven approach drawing from implementation science and sustainability literature, can optimize the benefits of the intervention, prolong sustainability, and promote dissemination into other contexts [19].

## Methods/Design

### Aim and objectives

The overall aim of this study is to generate evidence on the implementation effectiveness and sustainability of the MAHMAZ project and reasons for variation in order to inform the interpretation of the outcomes of the main trial. The specific objectives of our study include:

1. To assess the degree to which each intervention component is implemented according to the project plan and to document any adjustments and/or adaptations.
2. To identify barriers and facilitators to implementation of the intervention components.
3. To assess the extent to which implementation of the Core Model is perceived as responsive to community standards of acceptability by both the community and the women compared to the standard of MWH care at control sites.
4. To assess the ways in which health facility-level factors change over the course of implementation in MAHMAZ sites compared to control sites, both during the intervention and after direct external support has ended.
5. To assess the ways in which service utilization patterns at both MWH and rural health centers change over time in MAHMAZ sites compared to control sites both during the intervention and after direct external support has ended.
6. To develop a contextual history of other factors that may influence both the implementation and the outcomes at the community, district, provincial and national levels.
7. To identify key features of the entrepreneurial strategies and management models developed to support MWH operations, and assess their immediate and sustained effectiveness.

### Study setting

The MAHMAZ cRCT is taking place in three districts in the Southern Province (Choma, Kalomo, and Pemba) and one district in Eastern Province (Nyimba) of Zambia. All study districts are primarily rural. Choma district has a total population of 247,860, with 76% living in rural areas. Kalomo, which has the largest rural population in Southern Province, has a total population of 258,570, with about 93% living in rural areas [20]. During the 2010 census Pemba was part of Choma district. Nyimba district has a total population of 77,359, with 91% living in rural areas [21].

A total of 22 health facilities in the districts were eligible for the intervention based on criteria to ensure a basic level of quality, including transfer time to nearby facilities that provide comprehensive emergency obstetric and newborn care (2 h or less), delivery volume (≥150 per year), and either 1) the ability to perform basic emergency obstetric and newborn care (BEmONC) signal functions (five out of seven), which are a set of interventions to care for the mother and newborn during intrapartum care [22] or 2) had at least one skilled attendant on staff, routinely practices active management of third stage of labor, and had no stock outs of oxytocin or magnesium sulfate in the preceding 12 months. The 20 facilities farthest from the referral hospital were selected and matched into pairs based on travel time to referral hospital and delivery volume. Pairs were then randomly assigned to the intervention or control group. Detailed information about the setting, randomization process, and selection criteria can be found in the trial protocol [10]. MWHs were also constructed at two of the five main referral hospitals in the study districts. The MWHs at referral hospitals do not include all of the intervention elements of the Core Model. They are being assessed separately and therefore are not detailed in this implementation protocol.

### Study design and conceptual framework

We will use a mixed-methods approach and collect longitudinal, cross-sectional data at both the MAHMAZ intervention rural health center sites (*n* = 10) and the matched control sites (n = 10) before and during MAHMAZ project implementation, and for a short period (approximately 4 months) after cessation of project support.

Our study is guided by an integrated framework drawing from the Consolidated Framework for Implementation Research (CFIR) by Damschroder et al [19] and the Conceptual Framework for Sustainability of Public Health Programs (the Sustainability Framework) put forward by Scheirer and Dearing [18]. CFIR synthesizes constructs from multiple key implementation frameworks that are hypothesized to influence implementation. The Sustainability Framework posits the relationships between factors affecting sustainability and sustainability outcomes within a broader socio-political environment. Because these frameworks address different but related key drivers of program success – implementation and sustainability – elements from each underpin our study. We discuss these frameworks in more detail in the analysis section below.

### Data collection activities

**Fig. 1:**
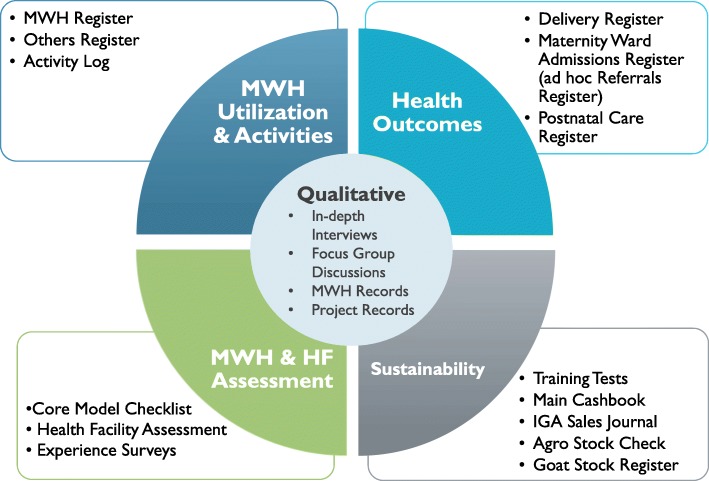
Routine implementation data by category

Figure 1 summarizes data collection tools (Fig. 1). The four categories of quantitative tools, represented in the outer circle, include those designed to assess MWH utilization and activities; facility delivery and health outcomes; fidelity and quality of the MWHs and the health facilities; and the potential for sustainability. Qualitative tools, represented in the inner circle (in-depth interviews [IDIs], focus group discussions [FGDs], MWH leadership records, and project records) serve to triangulate and provide depth and context to the quantitative data (Additional files 1, 2, 3, 4, 5, 6, 7, 8 and 9). Table 2 links the evaluation questions, data collection activities and data sources by objective. Table 3 describes each tool, its original data source, and the frequency of data collection. The maximum estimated sample size for this evaluation is 14,400 (Table 4). Qualitative data may have a lower sample size as we will stop during any given round when we reach saturation or predictability. The record review is estimated as utilization is likely to vary across sites. If we approach the maximum sample size because of underestimating utilization, we will amend the protocol.

**Table 2:** Objectives, Questions, Data Sources for the MAHMAZ Implementation Effectiveness Protocol

Objective	Evaluation Questions	Data Sources
1. To assess the degree to which each individual intervention is effectively implemented according to the project plan and to document any adjustments and/or adaptations	a. At intervention sites, what is the proportion of MWHs that meet each component of the Core Model? b. What proportion of intervention sites are operating according to the project standard operating procedures?	**Quantitative** MWH Register Others Register MWH Activity Log MWH Experience Survey Core Model Checklist **Qualitative** Project records
2. To identify barriers and facilitators to implementation of the interventions	a. What are the barriers and facilitators to implementation of the: 1. Core Model? 2. Governance and management models? 3. IGAs?	**Qualitative** IDI with MWH governance and management structures IDI with IGA staff/volunteers IDI with health facility/system staff
3 To assess the extent to which implementation of the Core Model is perceived as responsive to community standards of acceptability by both the community and the women.	a. How does satisfaction with staying at the MWH compare in intervention sites relative to control sites? b. To what degree are the intervention MWH perceived as responsive to community standards and needs? c. What are the essential features and characteristic of the MWH as perceived by both women and communities? d. What are continued barriers to accessing and utilizing MWH after the intervention?	**Quantitative** MWH Experience Survey **Qualitative** IDIs with MWH governance and management structures FGDs with pregnant/ recently delivered women (RDW), men with children < 1, community el ders, Safe Motherhood Action Group members (S MAGs)
4. To assess the ways in which health facility-level factors change over the course of implementation.	a. How does capacity to handle obstetric emergencies change over time at the facility? b. How do staff perceptions of care related to maternal health change over time? c. What role does the MWH have in shaping staff perceptions of maternal health care?	**Quantitative** Health Facility Assessment **Qualitative** IDI with health facility/system staff
5. To assess the ways in which service utilization patterns at both MWHs and rural health centers change over time.	a. How does the utilization of the MWH change over time? b. How do referral patterns, census and demographic of those utilizing health facilities change over time? c. What are the perceived changes in service utilization at both MWH and health facilities over time?	**Quantitative** Delivery Register Maternity Ward Admissions Register (for referrals) or ad hoc referral register Postnatal Care Register MWH Register **Qualitative** IDI with health facility/system staff IDI MWH governance and management structures Project records
6. To develop a contextual history of other factors that may influence both the implementation and the outcomes at the community, district, provincial and national levels.	a. What external factors may have influenced the implementation and outcomes observed? b. How does the external and policy environment of the health system influence the utilization and uptake of MWH for delivery?	**Qualitative** IDI with health facility/system staff IDIs with MWH governance and management structures FGDs with pregnant/ RDW women, men with children < 1, elders, SMAGs Project records
7. To identify key features of the entrepreneurial strategies and managements models developed to support MWH operations and to assess their immediate and sustained effectiveness.	a. What operational and financial systems are in place and functional at baseline? b. What is the contribution of each strategy in the overall financial sustainability of the MWH, and how does this change over time? c. Which strategies and managements models are perceived as viable for long-term sustainability? What differences exist between communities? d. What is the effect of each strategy/management model on the utilization of the MWH?	**Quantitative** MWH Main Cashbook IGA Sales Journal Monthly Goat Stock MWH Register **Qualitative** IDI with health facility/system staff IDI with MWH governance and management units FGDs with pregnant/ RDW, men with children < 1, elders, SMAGs Project records

**Table 3:** MAHMAZ Implementation Evaluation Data Collection Tools and Timing of Administration

Tool	Original Data Source	Description	Timing
Quantitative data collection tools			
Delivery and Neonatal Outcomes	Delivery Register at Health Facility	Extract key information about maternal and neonatal delivery outcomes	Monthly collection of previous calendar month
Referrals	Maternity Ward Admission Register at Health Facility	Extract information about referrals. In the absence of this register, the information will be extracted from the appropriate improvised register found at the health facility.	Monthly collection of previous calendar month
Postnatal Care	Postnatal Register at Health Facility	Extract key information about postnatal care utilization.	Monthly collection of previous 2 calendar months
MWH Register	MWH Register at MWH	Extract information about pregnant, waiting, and postpartum women using the MWH.	Monthly collection of previous 2 calendar months
Others Register	Others Register at MWH	Extract information about anyone not included in the MWH Register who are using the MWH.	Monthly collection of previous calendar month
Activity Log	Activity Log at MWH	Extract information about activities that occur at the mothers’ shelter.	Monthly collection of previous calendar month
Core Model Checklist	Observation of MWH and Consultation with MWH Management	Collect information on infrastructure, equipment and supplies, management function, linkages with the associated health facility, and educational services being offered at the MWH. Questions were designed to address each component of the Core Model that guides the overarching implementation for the MHA.	Monthly collection based on current observations
MWH Experience Survey	Women currently using the MWH	This is primarily a quantitative instrument that captures women’s general perceptions of and experience in the MWH (or other facility-affiliated structure in control sites) as related to the Core Model components; opportunity costs incurred while staying at the MWH; and intended future use.	Monthly
Health Facility Assessment	Health facility staff	This short tool will be used to assess the capacity of the health facility to manage obstetric complications.	Monthly
Qualitative data collection tools			
In-depth Interviews	1) Provincial & District Government 2) Health Facility Staff 3) MWH Governance 4) MWH Management 5) IGA	Elicit information on the challenges, strengths, and perceived impact of the maternity home on the health facility staff and perceived patient behavior in terms of utilization, bypassing, etc. Also elicit perceptions of MWH within the health system and changes in prioritizing, financing, and planning for MWHs. Themes will include perspectives on governance and management challenges and successes, progress toward financial and operational sustainability, perspectives on community ownership of the MWH, MWH responsiveness to community needs/acceptability, and ideas for improvements. Elicit information on challenges and successes of the IGAs and perceived impact of the IGAs on the MWHs.	Every 6 months
Focus Group Discussions	1) Pregnant or women with a child under 1 year 2) Men with a child under age 1 3) Community elders or mother-in-law 4) SMAG members	The FGDs will elicit perspectives on: 1) the quality of the mothers’ shelters, 2) community ownership, 3) barriers and facilitators to access to care, 4) thoughts on sustainability, 5) thoughts on ways to improve each domain.	Once a year in 2016, 2017, and 2018
IGA-specific data collection tools			
Main Cashbooks	Maternity Waiting Home Main Cashbook	Extract information about the revenue and expenses for the MWHs and the IGAs.	Monthly collection of previous calendar month
Sales Journals	IGA Sales Journal	Extract information about the items sold at each IGA.	Monthly collection of previous calendar month
Monthly Goat Stock	Monthly Goat Stock Taking Book	Extract information about the change in goat numbers each month at the Goat Rearing IGAs.	Monthly collection of previous calendar month

**Table 4:** Maximum Estimated Sample Size by Data Collection Method for the MAHMAZ Implementation Effectiveness Protocol

Method	Sample Size	Rationale
MWH Experience Survey	2880	A maximum of 6 women per site × 20 BEMONC sites X 24 months.
IDI with MWH Governance and Management Units	300	6 IDIs (3 governance members and 3 management members) per site X 10 BEmONC Intervention sites X 5 semi-annual cycles (July 2016–June 2018). This is the estimated sample size, but we will quit if we reach saturation or predictability during any given semi-annual cycle.
Focus Group Discussions	1200	10 participants per FGD X 4 FGDs site X 10 sites X 3 time points. This is the estimated sample size, but we will quit if we reach saturation or predictability during any given semi-annual cycle.
IDI with Health Facility/System staff	360	(3 IDIs per site × 20 BEmONC sites) + (3 IDIs per District/Province X 4 Districts) X 5 semi-annual cycles (July 2016–June 2018). This is the estimated sample size, but we will quit if we reach saturation or predictability during any given semi-annual cycle.
Record Review (health facility and MWH registers)	14,400	Data will be extracted monthly at all sites. We estimate 20 unique records per month x 20 BEmONC sites X 36 months.
TOTAL:	19,140	

#### Objective 1

In order to assess the degree to which each individual intervention was implemented according to the project plan and to document any adjustments and/or adaptations, we will extract data from MWH Registers, conduct MWH Experience Surveys and the Core Model Checklist, and review project records. On a monthly basis, we will systematically extract and aggregate data from the registers created for this project (MWH Register, Others Register, and Shelter Activity Log) at intervention and control health facilities, on MWH utilization and educational classes provided at the homes.

On a monthly basis, the Monitoring & Evaluation (M&E) research assistants will visit each site and will administer the MWH Experience Survey. This is a primarily quantitative instrument that captures women’s general perceptions of and experience in the MWH (or other facility-affiliated structure in control sites) as related to the Core Model domains; opportunity costs incurred while staying at the MWH; and intended future use. The MWH Experience Survey captures the domains of quality that the community members identified as important to them in the formative evaluation [9]. The survey captures whether or not a particular element of the Core Model: 1) was available to the waiting mother at the time of say; 2) was utilized by the waiting mother at the time of stay; and 3) the waiting mother’s perception of quality of each Core Model element. These data are important for monitoring whether or not the service is well-received by our population of interest and what changes could be made to improve implementation. At each site, study staff will use the MWH register to randomly select a sample of up to six women who have been at the MWH/existing structure for at least three consecutive nights on the day of the visit and have not previously participated in the survey.

We will implement a Core Model Checklist to determine the degree to which each of the sites is implementing elements of the Core Model and how standard of care compares at control sites. The Core Model Checklist includes the same domains and Core Model elements as the Experience Survey so as to facilitate comparison and be confident in our assessment of fidelity. The Core Model Checklist will be administered by study staff via an electronic data capture system installed on tablets on a monthly basis at the MWH and will collect information on infrastructure, equipment and supplies, functioning of the management structures, and linkages with the associated health facility. Study staff will observe the MWHs/existing structures and surrounding areas to assess quality and document any quality problems. Study staff will also document whether MWHs/existing structures are adhering to standard operating procedures and implementing elements of the Core Model.

Lastly, project records will be reviewed regarding construction for MWHs and IGAs, outfitting of MWHs and IGAs, timing of trainings, training attendance logs and materials covered. Additionally, general project mentorship activities will be reviewed for implementation timelines and how well they adhered to initial plans. Pre/post training tests provide documentation of the effectiveness of the governance, financial management, and IGA skills trainings. Final make-up of the governance and management, financial management, and IGA structures will be compared to the original plan, including documents developed through stakeholder participation, such as terms of reference for the governance committee, standard operating procedures for the MWH, and financial management guidelines.

#### Objective 2

We will use the qualitative data to identify barriers and facilitators to implementation. The study staff will conduct IDIs semi-annually with selected MWH governance committee members and MWH management unit members after the MWHs open (Additional files 3 and 4). These IDIs will be conducted in10 intervention sites, with at least 2 and up to 4 members per site per round (2 governance/2 management). The interviewer will use a semi-structured guide developed from the core frameworks to prompt respondents to discuss each area and probe responses. Questions will examine perspectives on governance and management challenges and successes, progress toward financial and operational sustainability, community ownership, responsiveness to community needs and acceptability, ideas for improvements, and how the IGAs are functioning.

We will also conduct semi-annual IDIs with IGA staff/volunteers starting after the IGAs become operational. Questions will gather perspectives on challenges and successes of the IGA, daily operations of the IGA, financial management structures, linkages with the MWH, and progress toward financial and operational sustainability of the MWH. These IDIs will be conducted in 10 intervention sites, with at least 1 and up to 2 members per site per round. The interviewer will use a semi-structured guide to prompt respondents to discuss each area and probe responses (Additional file 5).

Lastly, we will conduct IDIs with the health facility staff and district/provincial staff to understand challenges, strengths, and perceived impact of the Core Model on the health facility staff and perceived patient behavior in terms of utilization, bypassing, etc. At control sites, we will ask questions in similar domains, but as per the standard of care. We will also conduct IDIs among the district health staff to understand perceptions of MWHs generally, how they have impacted the health system, and changes in prioritizing, financing and planning for MWHs. We will also ask about general principles of governance and management of MWHs (Additional files 1 and 2).

#### Objective 3

We will use the MWH Experience Survey (see Objective 1), and semi-annual IDIs with governance committee and management unit members (see Objective 2) to assess the extent to which implementation of the Core Model is perceived as responsive to community standards of acceptability. In addition, at baseline (2016), midline (2017) and endline (2018), study staff will conduct FGDs among 1) pregnant or recently delivered women (RDW), 2) men with a child under age 1, 3) community elders/mothers-in-law, and 4) Safe Motherhood Action Group (SMAG) members, a cadre of community health worker. The FGDs are designed to elicit perspectives on: 1) the quality of the mothers’ shelters, 2) community ownership, 3) barriers and facilitators to access to care, 4) sustainability, and 5) ways to improve (Additional files 6, 7, 8 and 9). We will capture basic demographic information on each participant, including past use of an MWH.

The team will use a guide to prompt respondents to discuss each topic and probe responses. At control sites, FGD guides will be structured using the same domains but tailored to the standard of care.

#### Objective 4

We will assess changes to health facility-level factors over the course of implementation through the quantitative Health Facility Assessment tool and qualitative IDIs with health facility/systems staff. The Health Facility Assessment tool measures the capacity of the health facility to manage obstetric complications. Questions focus on infrastructure and equipment, including delivery and postnatal beds, the number and qualifications of staff, the ability of and frequency that staff have performed essential obstetric and newborn procedures, and stock outs of essential obstetric-related medicines. We will monitor capacity over time and identify if changes are associated with demand that may be facilitated by the implementation of the MWH. We anticipate respondents for the health facility assessment will be the health facility in-charge, or another staff with knowledge of facility capacity. To complete the assessment, project staff will consult facility registers and health facility staff. We will also conduct semi-annual IDIs with health facility staff and health system staff (see Objective 2).

#### Objective 5

We will use quantitative and qualitative data to assess how service utilization patterns at both MWH and rural health centers may change over time. We will routinely collect health facility data at the 20 sites beginning at the start of the calendar year, approximately 9 months prior to implementation of the intervention. On a monthly basis, study staff will extract key variables about delivery and neonatal outcomes, referrals and postnatal care from routine data systems including the Delivery Register, Maternity Ward Admission Register (or ad hoc referrals register), and Postnatal Care Register. We will also extract the MWH utilization from the MWH Register monthly from the start of the intervention at all 20 sites (see Objective 1). Secondly, using the Safe Motherhood Number (SMN), a unique code that a woman receives when visiting government facilities during her pregnancy, each woman will be tracked from admission to the MWH through delivery and postnatal visits, to understand utilization of MWH and health facility services and maternal and neonatal health outcomes. We will compare this information with data captured in Objective 1.

#### Objective 6

To develop a contextual history of factors that may have influenced implementation or outcomes at the community, district, provincial, and national levels, we will analyze qualitative data sources, including IDIs with health facility/systems staff (see Objective 2), IDIs with MWH governance and management structures (see Objective 2), and FGDs with relevant community members (see Objective 3). Throughout the project, we will also document, through project logs and meeting minutes, other events that occur that may affect the patterns of utilization or the effectiveness of our intervention, such as openings of new health facilities or redistricting of current ones.

#### Objective 7

To identify the key features of entrepreneurial strategies and management models developed to support the finances and operations of the Core Model, and to assess its effectiveness, we will use routinely collected monitoring data from registers, activity logs, training attendance logs, and the Core Model Checklist. From intervention sites, we will extract data from registers implemented by the project at the IGA (Main Cash book, Sales Journal and Monthly Goat Stock) to assess the revenue and expenses of the IGAs and MWH, quantity of products or services sold at the IGAs and stock at the goat rearing sites. For each type of IGA implemented, we will capture the costs of implementation, the MWH revenue over time from IGAs, community donations, or health facility contributions, and the proportion of MWH operating costs covered. Additionally, we will use program data to determine decisions made and changes occurring in MWH operating costs over time to understand what mix of operating costs and revenue streams create a financially sustainable MWH. We will triangulate findings with data from the semi-annual IDIs conducted with governance and management structures, IGAs staff/volunteers, and health facility staff (see Objectives 2 and 3).

For all qualitative data collection, all participants will be screened for eligibility and study staff will obtain informed consent. Study staff conversant in English and the appropriate local language, and trained in qualitative research methods and human subjects protection, will conduct the IDIs and FGDs. IDIs and FGDs will be recorded and transcribed verbatim, and translated to English when necessary.

### Inclusion criteria

Each data collection method will use its own set of inclusion and exclusion criteria as different participants will be enrolled depending in the method (Table 5).

**Table 5:** Respondent Inclusion and Exclusion Criteria by Data Collection Method

Method	Respondents	Inclusion Criteria	Exclusion Criteria
MWH Experience Survey	Recent users of the MWH	• Mother utilizing the maternity home for at least 3 consecutive nights • Mother utilizing the home for maternal health services (ANC, labor or PNC visit) • Currently pregnant or under 6-weeks post-partum • >/= 15 years of age (emancipated minor)	• Has responded to the survey at a previous visit for the same maternal health services (ANC, labor or PNC) • Utilizing the home for reasons unrelated to maternal health • Unwilling or unable to provide informed consent
In-Depth Interviews	MWH Governance Committee & Management Unit Members; IGA Officer and SMAG Tailor	• Is a current governance committee/management unit member at an intervention site OR is responsible for overseeing MWH operations at a control site OR is an IGA Officer OR is a SMAG • Is capable of responding to the domains • >/= 18 years of age	• Is not currently affiliated with governance or management of MWH any of the study sites • Unwilling or unable to provide informed consent
Focus Group Discussions	Pregnant or RDW, Male with child < 1 year, Elders/mother in-law (MIL), SMAGs or other potential SMAG member including traditional birth attendant (TBA), community health worker (CHW), or any individual currently volunteering at the health centre in an MCH capacity	• Respondent is a SMAG member or other potential SMAG member including TBA, CHW, or any individual currently volunteering at the health centre in an MCH capacity, Elder or MIL in a study catchment area • Or is a pregnant woman or with a child under the age of 1 • Or is a male with a child under the age of 1, • >/= 15 years of age	• Has previously participated in a FGD in the same year at another site or in another category (i.e.: SMAG and recently delivered) • Is not currently affiliated with any of the study sites • Unwilling or unable to provide informed consent
IDI with Health Facility/System staff	Health Facility Staff and Health System Staff	• Is a current health facility staff member at any one of the study sites (intervention, control, CEMONC) • Is knowledgeable in maternal health capacity at the health facility • Or is knowledgeable in health facility financial records • Or is a district or provincial health officer in the study districts • >/= 18 years of age	• Is not currently affiliated with any of the study sites • Unwilling or unable to provide informed consent
Record Review	User of the health system/MWH	• Is recorded in MWH Register, Others Register or Activity Log between January 2016 – December 2018. • Is recorded in BEmONC facility-based data: Delivery Register, Maternity Ward Admission Register, Postnatal Care Register, Facility Transfer Log, Ante-natal care register (where applicable) and other improvised registers as necessary between January 2016 – December 2018.	• Was recorded in MWH or health facility registers before January 2016 or after December 2018

## Data management

Study staff will oversee data collection, entry, management and storage. Three of the registers (Delivery, Postnatal, and Referrals) are existing instruments implemented by the Ministry of Health (MoH). All other instruments discussed in this protocol were developed and will be implemented by the study team. To ensure quality for both project records and MoH registers, study staff will obtain permission from the MoH at the district levels to provide mentorship to health facility staff who complete the registers. During monthly data extraction visits, study team members will make note of specific gaps in the completion of each instrument and provide mentorship to address the gap. Gaps will be followed up the following month to ensure they are not persisting. Additionally, there will be some duplication of demographics across registers to allow for triangulation in the event of missing data.

All quantitative data will be captured using SurveyCTO Collect Software (Version 2.212; Dobility, 2017) installed on password protected tablets. The electronic survey forms will be encrypted and uploaded to a secure server upon completion, only accessible by the study team. Records from individual women will be linked through matching identifiable data fields (such as SMN, name, village, age, gravida, parity, delivery date), then women will be assigned a unique ID and identifiable information will be stripped and stored in a separate password protected linking file, only accessible by the study staff. Digital recordings will be downloaded to a password protected computer and paper copies of qualitative notes will be kept in a locked cabinet until they are fully transcribed, at which point audio files will be deleted and notes shredded. The qualitative transcriptions will not contain identifying information, only a unique study identification number. Only the study analytic team will have access to the data.

## Analysis

### Analytic framework

As described earlier, our Core Model for the MWH is theory-driven using the CFIR framework. We will use this framework to analyze data related to implementation effectiveness. To assess the aspects of sustainability, we will use the Sustainability Framework [18]. We want to know which types of programs and outcomes continue after the cessation of direct external support and under what circumstances. Sustainability outcomes are thought to be affected by processes that happen throughout the different phases of a project, especially the earlier phases, and the context of which it is implemented (social, policy, and financial environment) [18]. This framework will guide analysis of how components of the project may be contributing to sustainability of the MWHs.

### Quantitative analysis

Quantitative analyses will be conducted in SAS version 9.4 (SAS Institute, Cary, NC). Time-series data from utilization records will be analyzed using an interrupted time series analysis. Detailed analysis of quantitative data for specific tools is described below:

#### MWH experience survey

Survey data will be analyzed by calculating descriptive statistics stratified by intervention/control sites. We will also look for differences in indicators within the matched pairs. Categorical variables will be compared between intervention and control groups using the chi-squared test if cell sizes are sufficient or Fisher’s exact test if cell sizes are small; continuous variables will be compared using t-tests if normally distributed or non-parametric Wilcoxon rank sum tests if distribution is non-normal.

#### Healthy facility assessment and core model checklist

The Health Facility Assessment will be analyzed to determine proportion of sites meeting the standards for signal functions and staffing, and the Core Model Checklist will be analyzed to determine the proportion of sites meeting Core Model domain requirements. Data will be stratified by intervention and control as well as district/province and compared over time.

### Qualitative analysis

Since we are using a repeated cross-sectional samples design, we will analyze each wave of data first, then analyze themes over time, connecting what has changed and how these changes might have occurred. We will conduct a content analysis of the IDI transcripts. Codes have been identified a priori according to the outline of the interview guides. We will then derive meaning from the coded data using the following steps: 1) familiarization; 2) identifying a thematic framework; 3) indexing; 4) charting; and 5) mapping and interpretation [23]. Qualitative data from IDIs and FGDs will be analyzed against the community-defined standards of acceptability from the formative evaluation [9]. Additional themes will be included if they emerge during analysis. Stratified by intervention and control site, we will review qualitative and quantitative data together to understand acceptability as measured by implementation fidelity of the Core Model, and findings from the impact evaluation.

## Ethics and dissemination

This study protocol received ethical approvals from the Boston University Medical Campus Institutional Review Board (IRB) and the ERES Converge IRB in Zambia. Written informed consent will be obtained by study staff and documented through signatures for all interviews and surveys. If the respondent is unable to provide a signature, the respondent will provide a thumbprint and an impartial, literate witness will sign as well after witnessing the informed consent process. Informed consent will not be obtained for the data extracted from the registers. The study staff ensures extracted data are kept private and confidential, and that they are de-identified as soon as women are linked across registers using the SMN number. Any adverse events and unanticipated problems will be reported to the in-country project director and communicated to the principal investigator. All adverse events and unanticipated problems will be reported to the ethical boards per policy and the protocol will be amended if necessary. Results will be disseminated among key stakeholders in Zambia, then through appropriate open-access channels.

## Discussion

Alongside the impact results of the cRCT, the findings from this implementation effectiveness and sustainability evaluation will be analyzed in order to interpret outcomes, identify mechanisms of impact and understand how variations in implementation affect these outcomes. We also aim to provide evidence of factors that may improve longer-term sustainability of MWH in this context. Results will be combined with detailed costing data being collected concurrently in order to also understand cost outcomes and cost-effectiveness. We aim to disseminate the combined results to donors, policymakers and program implementers in order to facilitate their future decision-making regarding the implementation of MWHs in Zambia. Results may be used as evidence for programmatic decisions by the government and supporting agencies in deciding to take this model to scale beyond the districts proposed for this project and to continue routine implementation of the model beyond the MAHMAZ project. In addition, this protocol may provide a guide for other researchers and evaluators to implement similar studies to assess implementation effectiveness and sustainability alongside more complex RCTs or quasi-experimental studies of program outcomes.

## Supplementary information

**Additional file 1.** In-Depth Interview Guide for Province and District Staff.

**Additional file 2.** In-Depth Interview Guide for Health Facility Staff.

**Additional file 3. ** In-Depth Interview Guide for Governance Committee.

**Additional file 4. **In-Depth Interview Guide for Management Unit.

**Additional file 5.** In-Depth Interview Guide for IGA Officer.

**Additional file 6.** Focus Group Discussion Guide for Pregnant and Recently Delivered Women.

**Additional file 7.** Focus Group Discussion Guide for Men with Children Under Age One.

**Additional file 8.** Focus Group Discussion Guide For Safe Motherhood Action Group/Community Health Worker.

**Additional file 9.** Focus Group Discussion Guide for Community Elders.

## Data Availability

The datasets used and analyzed during the current study are available from the corresponding author on reasonable request.

## Abbreviations

Africare/UM: Africare/University of Michigan
BEmONC: Basic emergency obstetric and newborn care
BU/RTC: Boston University/Right to Care Zambia
CEmONC: Comprehensive emergency obstetric and newborn care
CFIR: The consolidated framework for intervention research
cRCT: Cluster-randomized controlled trial
FGD: Focus group discussion
GRZ: Government of the Republic of Zambia
IDI: In-depth interview
IGA: Income generating activities
IRB: Institutional Review Board
M&E: Monitoring and Evaluation
MAHMAZ: Maternity Homes Access in Zambia Project
MHA: Maternity Homes Alliance
MoH: Ministry of Health
MSD: Merck Sharp and Dohme
MWH: Maternity waiting home
RDW: Recently delivered women
SMAG: Safe Motherhood Action Group
SMN: Safe motherhood number
SOP: Standard operating procedure
WHO: World Health Organization
