# Supplementary material for: Evaluating implementation effectiveness and sustainability of a maternity waiting homes intervention to improve access to safe delivery in rural Zambia: a mixed-methods protocol
Source: BMC Health Serv Res. 2020 Mar 12;20:191. doi: 10.1186/s12913-020-4989-x (PMC7068884; doi:10.1186/s12913-020-4989-x)
Supplement: Supplementary file 1 — Additional file 1. In-Depth Interview Guide for Province and District Staff. [file 12913_2020_4989_MOESM1_ESM.pdf]

SURVEY ID [ ]

Instrument ID: Form J1 ENGLISH

The MAHMAZ Project – Implementation Evaluation
In-depth Interview Guide with Provincial/District Health Staff

Target Audience:
Province and District Health Staff

Ensure respondent is a health staff member at district or provincial medical office, is knowledgeable about MCH issues (i.e. is the DMO/PMO, MCH Officer, EHT, or Public Health Officer) and is >=18 years of age.

Was written informed consent obtained for this interview?

☐ **YES (proceed with the interview)**

☐ **NO (STOP!** Thank the participant for their time. Do NOT proceed with the interview.)

Step 1: *Read the following statement. Note: All interviews with Provincial and District Staff will be conducted in English.*

Thank you for agreeing to participate in this interview. My name is ____________________. I will be asking you the questions and taking notes on the things you have to say. We want to understand in greater detail your perspectives on maternity waiting homes (MWHs) within the health system, as well as prioritization of financing and planning for MWHs. Please feel free to tell us only what you feel comfortable sharing. There are no right or wrong answers, so please be honest and help us to understand what is true for you and your colleagues, which include other health system staff. You can choose not to answer any questions.

Are you ready to begin**?**

Step 2: *Proceed to the interview guide. Please probe to obtain as in-depth and specific information you can.*

Interviewer Name ________________________

1. **Interview Date:**

DD MM YYYY

2. **Time Start:**

		:		
H	H		M	M

3. **Time Finish:**

		:		
H	H		M	M

Supervisor initials ___________________

SURVEY ID [ ]

Part 1: Respondent Demographics

Province: __________________
District (if applicable): ___________________

Interviewer: *"I'm going to start by asking you brief questions about your role."*

Q#	QUESTION	CODE	Response
100.	Respondent gender	Male (1)<br>Female (2)	
101	What is your current position?		
102	How long have you been un your current position?		
103	How long have you been working in the health system of your district/province?		

If interviewing a provincial officer, change "district" to "province" when asking the questions below.

Theme 1: Challenges and Strengths of Having an MWHs
Note to interviewer: This section is asking about both new and existing MWHs.

1a. In general, tell me about MWHs in your district? How are they ***functioning***?
Probe: How do the new MWHs compare to the old one?

1b. What is ***good about having MWHs*** in your district?
Probe: How do the new MWHs compare to the old one?

1c. What would you say are the ***drivers for these good things*** in your district?

1d. What is ***most challenging about having MWHs*** is your district?
Probe for:
- Construction, maintenance, management, monitoring or sustainability
- How do the new MWHs compare to the old ones?

1d. What ***has been done to address the challenges***?
Probe for:
- Levels: Hospital, District, Province
- Planning meetings, strategic planning, etc
- How do the new MWHs compare to the old one?

1d. What ***still needs to be done*** or what ***could be done better*** to address the challenges and sustain the successes?

Theme 2: Perceived impact of the MWHs on the health facility

"Now we are going to discuss how the newly constructed MWHs are impacting the health facility and the health facility staff."

2a. Please describe any impact – both positive and negative - that the newly constructed MWHs have had on ***health facility utilization*** **in your district**?

Probe for:

- Number of women being seen for ANC, Delivery, and PNC
- By-pass rates to CEmONC sites with MWHs

2b. Please describe any impact- both positive and negative – that the newly constructed MWHs have had on the ***operations at health facilities*** **in your district**?

Probe for:

- Task shifting
- Management and operations
- Financing structure (ie: IGAs)

2c. What is the ***impact on the health facility operations*** when you think only about the existing MWHs?

Theme 3: Perceived impact of the MWHs on the health system

3a. In the past six months, please explain any ***changes*** (positive or negative) that you have observed ***around prioritization of the MWHs*** **in your district.**

Probe for:

- Levels: Hospital, District, Province
- Changes in planning agenda or budgeting for MWHs

3b. Please explain any ***differences in prioritization*** between newly constructed MWHs and those existing MWHs at other facilities in your district/province.

Probe for: Planning, Budgeting, etc

Theme 4: Costs of MWHs

4a. What are the ***costs*** associated with having a ***newly constructed MWHs***?

- What are the costs for the health facilities and the district?

4b. What are the ***costs*** associated with having an ***existing MWHs***?

- What are the costs for the health facilities and the district?

4c. How does the health facility and district ***manage the costs***? Does the district budget currently have a ***line item*** for MWHs?

If yes:

i) How much is allocated to MWHs in the budget?

ii) How did you come to the decision to include a line item in the budget?

4d. Are you aware of any ***income generating activities*** associated with the MWHs? If so, please describe them for me.

4e. Has your district/province been ***approached by the MWHs' Governing Committee*** or anyone else about government contributions to the MWHs?

- Can you describe what was discussed or what contributions you have made?

Theme 5: Integration of MWHs into the Health System and GRZ initiative

"We will now discuss ownership of the MWHs and your vision for the long-term sustainability of the MWHs. This includes both financial and operation sustainability, such as sources of income, management, maintenance, utilization, etc."

5a. In your opinion, ***who owns MWHs in general***?

Probe for:

- Lands
- Income
- Material assets of the MWHS
- Does this differ between newly constructed and existing MWHs? Please explain.

5b. What are the ***roles*** that each of these people play to ensure success?

- Communities, women
- Health Facility Staff
- District Medical Office / Ministries
- Does this differ between newly constructed and existing MWHs? Please explain.

5c. Who is ***ultimately responsible for its success***? Please explain and give examples.

- To what degree is the ***government*** ultimately responsible for its success?
- Does this differ between newly constructed and existing MWHs? Please explain.

5d. How are we as partners ***integrating within the district plan***?

- Are we as partners working within the district plan or creating a separate system?
- What can we do to support the systems and not create parallel systems?
- Could anything be done better?

5e. What would you ***advise someone*** in your position in another district/province to do about integrating MWHs into their current system? What would you advise them NOT to do?

5f. What is the districts vision for ***long term sustainability*** of the MWHs?
Probe for: Financially, operationally

We have completed this interview. Is there anything else you would like to tell me?

"Thank you for your time. Please feel free to reach out if you think of anything else that may be helpful for us to know regarding MWHs."
