# Supplementary material for: Evaluating implementation effectiveness and sustainability of a maternity waiting homes intervention to improve access to safe delivery in rural Zambia: a mixed-methods protocol
Source: BMC Health Serv Res. 2020 Mar 12;20:191. doi: 10.1186/s12913-020-4989-x (PMC7068884; doi:10.1186/s12913-020-4989-x)
Supplement: Supplementary file 2 — Additional file 2. In-Depth Interview Guide for Health Facility Staff. [file 12913_2020_4989_MOESM2_ESM.pdf]

SURVEY ID

Instrument ID: Form J3 ENGLISH

The MAHMAZ Project – Implementation Evaluation
In-depth Interview Guide with Health Facility Staff

Target Audience:
BEmONC Health Facility Staff

Was written informed consent obtained for this interview?

☐ **YES**

☐ **NO – STOP!** Thank the participant for their time. Do NOT proceed with the interview.

Step 1: *Read the following statement. Please repeat the statement translated into the local language based on primary languages.*

Thank you for agreeing to participate in this interview. My name is ______________________. I will be asking you the questions and taking notes on the things you have to say. We want to understand in greater detail your perspectives on maternity waiting homes (MWHs) and utilization of health services at your facility. Please feel free to tell us only what you feel comfortable sharing. There are no right or wrong answers, so please be honest and help us to understand what is true for you and your colleagues, which include other health system staff. You can choose not to answer any questions.

Are you ready to begin?

Step 2: *Proceed to the interview guide. Please probe to obtain as in-depth and specific information you can.*

Interviewer Name _______________

1. **Interview Date:**

				**2**	**0**	**1**	

DD MM YY

2. **Time Start:**

		:		
H	H		M	M

3. **Time Finish:**

		:		
H	H		M	M

Supervisor initials ___________________

SURVEY ID

Part 1: RespondentDemographics

District: ________________________
Health facility: __________________

Interviewer: *"I'm going to start by asking you brief questions about your role."*

Q#	QUESTION	CODE	Response
100.	Respondent gender	Male (1)<br>Female (2)	
101	What is your current position?		
102	How long have been in your current position?		
103	How long have you been working in the health system of your district/province?		

Theme 1: Challenges and Strengths of having a functioning MWHs

1a. Think about the MWHs (or alternative waiting space for pregnant women) at this facility. Please describe it for me.
Probe for: Structure, quality, assets (beds, linen, cooking supplies, etc)

1b. Have there been any recent changes or improvements to the MWH?

1c. What is ***good about having a MWHs*** at your health facility?

1d. What would you say are the ***drivers for these good things*** about the MWHs?

1e. What are the ***challenges*** of your MWHs?

1f. What can be done to ***address these challenges***?
Probe for:

- Health facility level
- District level
- Provinicial level
- Planning meeting, strategic planning, etc

Theme 2: Impact of the MWHs on health facility operations & staff

2a. What is the ***role*** of each actor in ensuring the success of the MWHs?
Ask about:

- Management Unit **(Intervention sites only!)**
- Governance Committee **(Intervention sites only!)**
- Community

2b. What ***responsibilities do health facility staff have*** towards the operations of the MWHs? How do these responsibilities ***impact the health facility staff*** and their workloads?
Probe for:

- Management of MWHs daily operations
- Checking in on women at the MWHs

2c. In the last 6 months, have there been any ***changes in utilization*** of the health facility? Please explain. How have these changes ***impacted the health facility and staff***?
Probe for:

- For ANC
- For Delivery
- For PNC
- Is this because of the MWHs?

2d. In the last 6 months, have there been any ***changes in referal patterns*** at the health facility. Please explain. How have these changes ***impacted the health faclity*** and staff?
Probe for:

- Is this because of the MWHs?

Theme 3: HIV service delivery and the MWHs

3a. What services are offered at the health facility for ***HIV+ pregnant, delivering, and postnatal women?*** Please be specific

3b. How, if at all, does the ***MWHs affect how HIV+ women*** utilize these services?
Probe for:

- Pregnant
- Delivering
- Postpartum

3c. How, if at all, can the ***MWHs be used to provide HIV services*** to waiting women (for delivery or postnatal stays)?

Theme 4: Perceptions of costs associated with the MWHs

4a. What are the ***costs*** associated with having a functioning MWHs?

- Are there (financial) costs to the health facility that are as a result of having a functioning MWHs?
- If yes, how is the facility managing the extra costs?

4b. Is there a ***line item*** in the health facility budget for the MWHs?
If yes:
i) How much is allocated to the MWHs in the budget and what is it intended to be spent on?

ii) How did you come to the decision to include a line item in the budget?
Probe for: any advocacy from the governance committee towards funding the MWHs?

iii) What adjustments have been made to the facility budget to allocate some money towards the running of the MWHs? Has there been cuts to other line items? If so, what cuts have been made?

4c. Was any of the ***money provided by the district spent on the MWHs***, even if it wasn't originally budgeted for the MWHs?
If yes, what was the money spent on?

4d. Are you aware of any ***income generating activities*** that the MWHs is engaged in?
If yes, please describe them for me. How are they ***functioning***?
Probe for:

- Keyhole gardens
- Tailoring
- Income generating activities

Theme 5: Ownership & Sustainability of the MWHs
"We will now discuss your vision for the long term sustainability of the MWHs. This includes both financial and operation sustainability, such as sources of income, management, maintenance, utilization, etc."

5a. In your opinion, ***who owns the MWHs***?

- Who owns the lands?
- Income?
- Material assets of the MWHs?

5b. What would you do to ***improve the support*** for MWHs?

- Community support
- Health facility support

5c. What is your vision for the ***long term sustainability*** of the MWHs?

Probe for: financial and operational sustainability, maintenance and management

- What actions are involved in reaching this vision?
- **For Implementation Sites Only:** As a project, what can we do to facilitate reaching that vision?

We have completed this interview. Is there anything else you would like to tell me?

"Thank you for your time. Please feel free to reach out if you think of anything else that may be helpful for us to know regarding MWHs."
