# Supplementary material for: Evaluating implementation effectiveness and sustainability of a maternity waiting homes intervention to improve access to safe delivery in rural Zambia: a mixed-methods protocol
Source: BMC Health Serv Res. 2020 Mar 12;20:191. doi: 10.1186/s12913-020-4989-x (PMC7068884; doi:10.1186/s12913-020-4989-x)
Supplement: Supplementary file 3 — Additional file 3. In-Depth Interview Guide for Governance Committee. [file 12913_2020_4989_MOESM3_ESM.pdf]

SURVEY ID

Instrument ID: Form J4 ENGLISH

The MAHMAZ Project – Implementation Evaluation
In-depth Interview Guide with Governance Committee Members

Target Audience:

Governance Committee Members (chair or someone on the executive board and other member of the committee. One of the two participants must be female)

Was written informed consent obtained for this interview?

☐ **YES**

☐ **NO – STOP!** Thank the participant for their time. Do NOT proceed with the interview.

Step 1: *Read the following statement. Please repeat the statement translated into the local language based on primary languages.*

Thank you for agreeing to participate in this interview. My name is ____________________. I will be asking you the questions and taking notes on the things you have to say. We want to understand in greater detail your perspectives on governing and managing the maternity waiting homes (MWHs) in your community. Please feel free to tell us only what you feel comfortable sharing. There are no right or wrong answers, so please be honest and help us to understand what is true for you and your colleagues, which include other governance committee and management unit members. You can choose not to answer any questions.

Are you ready to begin?

Step 2: *Proceed to the interview guide. Please probe to obtain as in-depth and specific information you can. Note that we are asking the same questions to the governance members as we are to the management units. This is deliberate as we want to understand the same issues from different perspectives.*

Interviewer Name _______________

1. **Interview Date:**

DD MM YYYY

2. **Time Start:**

		:		
H	H		M	M

3. **Time Finish:**

		:		
H	H		M	M

Supervisor initials __________________

SURVEY ID [ ]

Province: ___________________
District: ____________________
HFCA Name: ______________________
HFCA ID: _________________________

Part 1: Respondent Demographics

Interviewer: *"I'm going to start by asking you brief questions about your role."*

Q#	QUESTION	CODE	Response
100.	Respondent gender	Male (1)<br>Female (2)	
101.	Occupation	Farmer/agricultural camp officer (1)<br>Health Facility Staff/SMAG/Midwife (2)<br>Clergy (3)<br>Teacher (4)<br>District/traditional government rep (5)<br>Business man/woman (6)<br>Social worker (7)<br>Housewife (8)<br>Other (9) Please specify_________	
102.	Have you ever attended school?	YES (1)<br>NO (0)<br>DON'T KNOW (97)	[ ][ ]<br>If (0) or (97), skip to #104.
103.	What is the highest grade you completed?	*Write grade level (i.e.: 03 for grade 3).*<br>*If <1 year completed, write down 00.*<br>*If >12 years completed, write down 13.*<br><br>DON'T KNOW (97)	[ ][ ]
104.	How old were you at your last birthday?	Please write age in the box.	[ ][ ]
105.	What is your role on the MWH Governance Committee?	Chairperson (1)<br>Secretary (2)<br>Treasurer (3)<br>General Member (4)<br>Other (5) Please specify__________<br>Don't know (97)	
106.	For how long have you served in this role?	Write in the years and months.<br>*If less than 1 year, enter 00.*	___________ years<br><br>___________ months

Theme 1: Challenges and Strengths of Having a MWH

1a. In general, tell me how the MWH is ***functioning***?
Probe for:

- Infrastructure, supplies, safety, comfort, space
- Registers
- Check-ins by health staff
- Classes, education, recreational activities

1ai. Tell me about daily maintenance of the MWHs (cleaning and watering of the gardens)

Probe for:

- Who cleans the homes?
- Who waters the gardens?

1aii. Do women complain about cleaning the homes and watering the gardens?

If yes, what do they say? How have you handled these complaints?

1b. Who in your community usually ***uses MWHs the most***? Why? Who in your community ***uses MWHs the least***? Why?

1c. What is ***good about having an MWH at your health facility***?
Probe for:

- Infrastructure, supplies, safety, comfort, space
- Registers
- Check-ins by health staff
- Classes, education, recreational activities

1d. What would you say are the ***drivers for these good things*** about the MWH?

1e. What have you noticed are the ***biggest challenges*** of your MWH?
Probe for:

- Infrastructure, supplies, safety, comfort, space
- Registers
- Check-ins by health staff
- Classes, education, recreational activities

1f. What has been done to ***address the challenges***? What still needs to be done?
Probe for:
- Levels: Management unit, Governance committee, Health Facility, District, Province
- Planning meeting, strategic planning, etc

Theme 2: Governance Committee

2a. From your perspective, what is the ***role of the MWH Governance Committee***?
i. ***Has this role changed over time?***
ii. Has the make-up of the committee changed over time?

2b. From your perspective, what is the ***role of the MWH Management Unit***?
i. ***Has this role changed over time?***

2c. Please tell me about the ***meetings*** the Governance Committee has, if any.
i. ***How often*** does the Governance Committee have meetings? What do you discuss at meetings?
ii. Are meetings useful? Why/why not?
iii. Which members attend the meetings regularly?

2d. Can you tell me about ***women's roles*** on the governance committee?
Probe for:
- Governance committee responsibilities
- Participation in meetings

2e. How does the Governance Committee and Management Unit discuss issues or ***communicate*** with each other?
i. What types of issues do you discuss together?
ii. How frequently?
iii. Tell me about a discussion.

2f. Can you describe your experience with ***sharing your opinions*** on the Governance Committee (during meetings and in other instances)?
i. Do you feel your opinions matter in the Governance Committee? Why/why not?

2g. Has the Governance Committee experienced any ***conflict*** among its members?
i. Can you tell me about a time when conflict arose?
ii. What was the conflict?
iii. How did you ***manage the conflict***?

2h. Has there been any ***conflict*** between the Governance Committee and Management Unit?

i. Can you tell me about a time when conflict arose?
ii. What was the conflict?
iii. How did you ***manage the conflict***?

2i. Please explain ***your role*** on the Governance Committee? Are there any challenges in your role?

i. Do you feel that you have the knowledge, skills, and resources to do what is being asked of you? Why/why not?

Theme 3: Perspectives on Governance Challenges and Successes

"We will now talk specifically about the governance and management systems of this MS. "

3a. What do you think are the ***qualities of a well-managed MWH***? Does your MWH have these qualities? Why/why not?

Probe for:

- Systems of the daily operations of the MWH
- Staff capacity

3b. What ***model of management*** is in place at this MWH?

i. ***What is working well*** with this model?
ii. ***What is not working well***?
iii. Have you made any ***changes*** to the management model in the past six months? If yes, what was the change and why?

3c. What ***systems*** do you have in place for ***prioritizing women*** living more than 10km away?

3d. What ***systems*** do you have in place for ***fast-tracking women*** staying at the MWH to ANC and PNC services offered at the health facility?

3e. What ***systems*** do you have in place for women to give ***feedback*** on their mothers' shelter experience *(Note to interviewer: separate from the experience survey)*

3f. In instances where something is not working well with the systems in place at the shelter, what do you think would improve this?
Probe for:
- Changes in policies and rules
- Organizational planning with MU

3g. In general, what would you ***advise someone*** who is just starting on a MWH governance committee?

Theme 4: Perspectives on Roles and Responsibilities of Health Facility and Community

4a. Please explain how, if at all, your MWH is ***linked with the health facility***.
i. Do health facility staff have specific responsibilities related to the MWH? Do ***health facility staff check in*** with the women staying at the MWH?
ii. How has this linkage been successful?
iii. What challenges have you experienced?
iv. What still needs to be done to link the MWH with the health facility?

4b. What would you ***advise someone in your position*** at another health facility to do about linking the MWH to their health facility? What would you ***advise them NOT to do***?

4c. Does the ***community contribute towards maintenance*** and/ or upkeep of the MWH? In what ways?
Probe for: drawing water, slashing, sweeping, weeding, cash, food, etc.

4d. Have community members made any ***monetary or in-kind contributions to the MWH*** since it was constructed?
i. What was contributed?
Probe for: building materials, money or maize from each household/village
ii. When were these contributions first made?
iii. How frequently have they been made since?
iv. Is there a plan for further contributions?
v. If maize (or any other in-kind contributions) was contributed, what was done with the maize?
Probe for: sold for money or provided to the women for nshima

4d. What would you do to ***improve the support*** for MWH?
i. What can the community do to better support the MWH?
ii. What can the HF do to better support the MWH?

Theme 5: Costs and Sources of Income

5a. What are the ***costs*** associated with keeping the MWH operational?
Probe for: Routine costs, maintenance

5b. Tell me ***how your MWH covers those costs***?

5c. In the last 6 months, did the MWH have any ***maintenance needs***?
Probe for:
- If yes, what needed maintenance?
- How was this fixed and who fixed it?
- Where did the funds for maintenance come from?

5d. What are the ***sources of income*** that this MWH has? Can you describe each of them?
Probe for: Health facility contribution, Income Generating Activities, community contributions

5e. For each source of income mentioned above, ***how much profit*** do you think will be made each month?
- Will this profit be sufficient to meet the MWH operational costs?
- Why/why not?

5f. How do you plan to ***allocate the funds generated***?

5g. How often do you ***review financial reports*** for the MWH? What do you look for when reviewing reports?

Theme 6: Perspectives on Ownership & Sustainability of the MWH

6a. In your opinion, ***who owns the MWH***?
- Who owns the lands?
- Income?
- Material assets of the MS?

6b. Who is ***ultimately responsible for its success***? Please explain and give examples.

6c. What would you ***advise the government*** to do to better link the MWH with the overall health system?

6d. What is your vision of the ***long-term sustainability*** of the MWH? You can take your time to think about this.
Probe for: Financial, operational, management, and maintenance

We have completed this interview. Is there anything else you would like to tell me?

"Thank you for your time. Please feel free to reach out if you think of anything else that may be helpful for us to know regarding the MWH."
