# Supplementary material for: Evaluating implementation effectiveness and sustainability of a maternity waiting homes intervention to improve access to safe delivery in rural Zambia: a mixed-methods protocol
Source: BMC Health Serv Res. 2020 Mar 12;20:191. doi: 10.1186/s12913-020-4989-x (PMC7068884; doi:10.1186/s12913-020-4989-x)
Supplement: Supplementary file 4 — Additional file 4. In-Depth Interview Guide for Management Unit. [file 12913_2020_4989_MOESM4_ESM.pdf]

SURVEY ID [ ]

Instrument ID: Form J5 ENGLISH

The MAHMAZ Project – Implementation Evaluation
In-depth Interview Guide with Management Unit Members

Target Audience:

Management Unit Members

Was written informed consent obtained for this interview?

☐ **YES**

☐ **NO – STOP!** Thank the participant for their time. Do NOT proceed with the interview.

Step 1: *Read the following statement. Please repeat the statement translated into the local language based on primary languages.*

Thank you for agreeing to participate in this interview. My name is ____________________. I will be asking you the questions and taking notes on the things you have to say. We want to understand in greater detail your perspectives on governing and managing the maternity waiting homes (MWHs) in your community. Please feel free to tell us only what you feel comfortable sharing. There are no right or wrong answers, so please be honest and help us to understand what is true for you and your colleagues, which include other governance committee and management unit members. You can choose not to answer any questions.

Are you ready to begin?

Step 2: *Proceed to the interview guide. Please probe to obtain as in-depth and specific information you can. Note that we are asking the same questions to the governance members as we are to the management units. This is deliberate as we want to understand the same issues from different perspectives.*

Interviewer Name _______________

1. **Interview Date:**

DD MM YYYY

2. **Time Start:**

[ ][ ] : [ ][ ]

H H **M M**

3. **Time Finish:**

[ ][ ] : [ ][ ]

H H **M M**

Supervisor initials __________________

SURVEY ID

Part 1: Respondent Demographics

Interviewer: *"I'm going to start by asking you brief questions about your role."*

Province: ___________________
District: ___________________
HFCA Name: ______________________
HFCA ID: _________________________

Q#	QUESTION	CODE	Response
100.	Respondent gender	Male (1)<br>Female (2)	
101.	Occupation	Farmer/agricultural camp officer (1)<br>Health Facility Staff/SMAG/Midwife (2)<br>Clergy (3)<br>Teacher (4)<br>District/traditional government rep (5)<br>Business man/woman (6)<br>Social worker (7)<br>Housewife (8)<br>Other (9) Please specify________	
102.	Have you ever attended school?	YES (1)<br>NO (0)<br>DON'T KNOW (97)	If (0) or (97), skip to #104.
103.	What is the highest grade you completed?	*Write grade level (i.e.: 03 for grade 3).*<br>*If <1 year completed, write down 00.*<br>*If >12 years completed, write down 13.*<br><br>DON'T KNOW (97)	
104.	How old were you at your last birthday?	Please write age in the box.	
105.	What is your role on the MWH Management Unit?	Health facility staff (1)<br>Paid volunteer (2)<br>Volunteer (3)<br>Other (4) Please specify_________<br>Don't know (97)	
106.	Are you the MWH Chairperson?	Yes (1)<br>No (0)<br>Don't know (97)	
107.	For how long have you served in this role?	Write in the years and months.<br>*If less than 1 year, enter 00.*	____________ years<br><br>____________ months

Theme 1: Challenges and Strengths of Having a MS

1a. In general, tell me how the MWH is ***functioning***?
Probe for:

- Infrastructure, supplies, safety, comfort, space
- Registers
- Check-ins by health staff
- Classes, education, recreational activities

1ai. Tell me about daily maintenance of the shelters (cleaning and watering of the gardens)

Probe for:

- Who cleans the shelter?
- Who waters the gardens?

1aii. Do women complain about cleaning the shelter and watering the gardens?

If yes, what do they say? How have you handled these complaints?

1b. Who in your community usually ***uses the MWH the most***? Why? Who in your community ***uses the MWH the least***? Why?

1c. What is ***good*** about having a MWH at your health facility?
Probe for:

- Infrastructure, supplies, safety, comfort, space
- Registers
- Check-ins by health staff
- Classes, education, recreational activities

1d. What would you say are the ***drivers for these good things*** about the MWH?

1e. What have you noticed are the ***biggest challenges*** of your MS?
Probe for:

- Infrastructure, supplies, safety, comfort, space
- Registers
- Check-ins by health staff
- Classes, education, recreational activities

1f. What has been done to ***address the challenges***? What still needs to be done?
Probe for:

- Levels: Management unit, Governance committee, Health Facility, District, Province
- Planning meeting, strategic planning, etc

Theme 2: Management Unit

2a. From your perspective, what is the ***role of the MWH Governance Committee***?

- Has this role ***changed*** over time?

2b. From your perspective, what is the ***role of the MWH Management Unit***?

- Has this role ***changed*** over time?

2c. ***How many people*** make up the Management Unit at this MWH?

If MU has more than one person, ask:

i) Does a ***duty rota*** exist at this mothers' shelter?
- If yes, how is the rota used? Is the rota followed by management unit members?

ii) Does the Management Unit hold ***meetings***? Please tell me about them.
- ***How often*** does the Management Unit have meetings?
- What do you discuss at meetings?
- Are meetings useful? Why/why not?
- Which members attend the meetings regularly?

2e. Has the Management Unit experienced any ***conflict*** among its members?

- Can you tell me about a time when conflict arose?
- What was the conflict?
- How did you ***manage the conflict***?

2d. How does the Governance Committee and Management Unit discuss issues or ***communicate*** with each other?

- What types of issues do you discuss together?
- How frequently?
- Tell me about a discussion.

2f. Has there been any ***conflict*** between the Governance Committee and Management Unit?

- Can you tell me about a time when conflict arose?
- What was the conflict?
- How did you ***manage the conflict***?

2g. Please explain ***your role*** on the Management Unit? Are there any challenges in your role?

- Do you feel that you have the knowledge, skills, and resources to do what is being asked of you? Why/why not?

Theme 3: Perspectives on Management challenges and successes

"We will know talk specifically about the governance and management systems of this MWH. "

3a. What do you think are the ***qualities of a well-managed MWH***? Does your MWH have these qualities? Why/why not?
Probe for:

- Systems for the daily operations of the MWH
- Staff capacity

3b. Tell me ***how your MWH is managed*** on a day-to-day basis?
Probe for:

- Registration, orientation processes, discharge
- Cleaning and maintenance
- Inventory and record keeping
- Facility check-ins

3c. In instances where something is not working well, what do you think would ***improve the systems at the MWH***?

- Changes in policies and rules
- Organizational planning with MU

3d. What would you ***advise someone*** who is just starting to manage a MWH at another facility?

Theme 4: Perspectives on Roles and Responsibilities of HF and Community

4a. Please explain how, if at all, your MWH is ***linked with the health facility***.
- Do health facility staff have specific responsibilities related to the MWH? Do ***health facility staff check in*** with the women staying at the MWH?
- How has this linkage been successful?
- What challenges have you experienced?
- What still needs to be done to link the MWH with the health facility?

4b. What would you ***advise someone in your position*** in another district or province to do about linking the MWH to their health facility? What would you ***advise them NOT to do***?

4c. Does the ***community contribute towards maintenance*** and/ or upkeep of the MWH? In what ways?
Probe for: Drawing water, slashing, sweeping, weeding, cash, food, etc.

4d. Have community members made any ***monetary or in-kind contributions to the MWH*** since it was constructed?

i. What was contributed?
   Probe for: building materials, money or maize from each household/village
ii. When were these contributions first made?
iii. How frequently have they been made since?
iv. Is there a plan for further contributions?
v. If maize (or any other in-kind contributions) was contributed, what was done with the maize?
   Probe for: sold for money or provided to the women for nshima

4e. What would you do to ***improve the support*** for MWH?
- What can the community do to better support the MWH?
- What can the health facility do to better support the MWH?

Theme 5: Perspectives on Ownership & Sustainability of the MS

5a. In your opinion, ***who owns the MWH***?
- Who owns the lands, income, and material assets of the MWH?

5ai. Who is ***ultimately responsible for its success***? Please explain and give examples.

5b. What would you ***advise the government*** to do to better link the MWH with the overall health system?

5c. What is your vision of the ***long-term sustainability*** of the MWH? You can take your time to think about this.
Probe for: Financial, operational, management, and maintenance

We have completed this interview. Is there anything else you would like to tell me?

"Thank you for your time. Please feel free to reach out if you think of anything else that may be helpful for us to know regarding MWH."
