# Supplementary material for: Evaluating implementation effectiveness and sustainability of a maternity waiting homes intervention to improve access to safe delivery in rural Zambia: a mixed-methods protocol
Source: BMC Health Serv Res. 2020 Mar 12;20:191. doi: 10.1186/s12913-020-4989-x (PMC7068884; doi:10.1186/s12913-020-4989-x)
Supplement: Supplementary file 5 — Additional file 5. In-Depth Interview Guide for IGA Officer. [file 12913_2020_4989_MOESM5_ESM.pdf]

SURVEY ID

Instrument ID: Form J6 ENGLISH

The MAHMAZ Project – Implementation Evaluation
In-depth Interview Guide with Income Generating Activity Officer

Target Audience:

IGA Officers and IGA Sub-Committee Member

Was written informed consent obtained for this interview?

☐ **YES**

☐ **NO – STOP!** Thank the participant for their time. Do NOT proceed with the interview.

Step 1: *Read the following statement. Please repeat the statement translated into the local language based on primary languages.*

Thank you for agreeing to participate in this interview. My name is ____________________. I will be asking you the questions and taking notes on the things you have to say. We want to understand in greater detail your perspectives on how the income generating activity (IGA) is functioning and its linkages with the maternity waiting home (MWH). Please feel free to tell us only what you feel comfortable sharing. There are no right or wrong answers, so please be honest and help us to understand what is true for you and your colleagues, which include other governance committee and management unit members. You can choose not to answer any questions.

Are you ready to begin?

Step 2: *Proceed to the interview guide. Please probe to obtain as in-depth and specific information you can. Note that we are asking the same questions to the governance members as we are to the IGA officers. This is deliberate as we want to understand the same issues from different perspectives.*

Interviewer Name _______________

1. **Interview Date:**

DD MM YYYY

2. **Time Start:**

		:		
H	H		M	M

3. **Time Finish:**

		:		
H	H		M	M

Supervisor initials ___________________

SURVEY ID | |

Part 1: Respondent Demographics

Interviewer: *"I'm going to start by asking you brief questions about your role."*

Province: __________________
District: __________________
HFCA Name: _____________________
HFCA ID: _________________________

Q#	QUESTION	CODE	Response
100.	Respondent gender	Male (1)<br>Female (2)	
101.	Occupation	Farmer/agricultural camp officer (1)<br>Health Facility Staff/SMAG/Midwife (2)<br>Clergy (3)<br>Teacher (4)<br>District/traditional government rep (5)<br>Business man/woman (6)<br>Social worker (7)<br>Housewife (8)<br>Other (9) Please specify________	
102.	Have you ever attended school?	YES (1)<br>NO (0)<br>DON'T KNOW (97)	If (0) or (97), skip to #104.
103.	What is the highest grade you completed?	*Write grade level (i.e.: 03 for grade 3).*<br>*If <1 year completed, write down 00.*<br>*If >12 years completed, write down 13.*<br><br>DON'T KNOW (97)	[ ][ ]
104.	How old were you at your last birthday?	Please write age in the box.	[ ][ ]
105.	What is your role in the IGA?	IGA Officer (1)<br>IGA Sub-Committee Member (2)<br>GC Treasurer (3)<br>Other (4) Please specify.<br>Don't know (97)	
106.	For how long have you served in this role?	Write in the years and months.<br>*If less than 1 year, enter 00.*	____________ years<br><br>____________ months
107.	Which IGA is associated with this MWH?	Agro-dealership (1)<br>Goat Rearing (2)<br>Hammer mill (3)	

Theme 1: Functional Aspects of the IGA

"We will begin by talking about the strengths and challenges of having an IGA in general. "

1a. Please tell me about your ***IGA in general***. We will go into more detail about each piece throughout the interview.

i. What is it for?
ii. What is the building like? Is it built appropriately for the IGA?
iii. How is it managed?
iv. Tell me about the sales?

1b. Can you tell me a story about ***something going well*** with the IGA? What was involved in making this good thing happen? Anything else that is going well?

Probe for:

- Community using the IGA
- Meeting a community need
- Profitability of the business
- Sales

1c. Can you tell me a story about ***something that is not going well***? Please provide a specific example. What was involved? Anything else that is not going well?

Probe for:

- Management or daily operations
- Stocking of items
- Handling of money (any theft?)
- Use of the business by the community

1d. What are the ***main challenges*** you face with the IGA?

1e. How have you ***addressed these challenges*** or how could you?

1f. From your perspective, what is the ***role of the IGA Officer***(s)?

i. Is this one person or multiple?
ii. What are their responsibilities?
iii. Has this role changed over time? How?
iv. How could the IGA Officer role be better?

1g. From your perspective, what is the ***role of the IGA Sub-Committee***?

i. Who is on the IGA Sub-Committee?
ii. What are their responsibilities?
iii. Has this role changed over time?
iv. How could the role of the IGA Sub-Committee be better?

1h. For ***your role in this IGA***, do you feel that you have the knowledge, skills, and resources to do what is being asked of you? Why or why not?

- What could make you better prepared for your job?

Theme 2: Operational Aspects of the IGA

"We will now talk specifically about how the IGA is operating. "

2a. Please tell me about what you keep in ***stock at the IGA*** to keep it running?
Probe for:

- For Agrodealership: Agro products (seed, fertilizer, chemicals), Birthing supplies (razor, plastic, jik, cotton thread), Baby clothes, Other
- For Hammermill: Fuel
- For Goat Rearing: Goats, medicines, feed, chemicals

2b. Are all of these ***items in stock today***? For the items not currently in stock today, why or why not?
Probe for:

- For Agrodealership: Agro products (seed, fertilizer, chemicals), Birthing supplies (razor, plastic, jik, cotton thread), Baby clothes, Other
- For Hammermill: Fuel
- For Goat Rearing: Goats, medicines, feed, chemicals

For the Agrodealership & Hammermill (2c)
2c. How can you tell if you have ***enough stock***? What do you do to keep track of this? Whose responsibility is it to track this?

For the Goat Rearing (2d-2e)
2d. How can you tell if your ***herd is healthy***? How do you keep them healthy?

2e. Tell me about a ***situation when some goats were not well***. How did you manage this situation?

2f. How often is ***the IGA open***?

i. Would customers want it open more or less?
ii. Has the IGA been closed or open as usual recently? Why or why not?

2g. How are the ***sales at the IGA***? Please provide more detail about each type of item.
Probe for:

- For Agrodealership: Agro products (seed, fertilizer, chemicals), Birthing supplies (razor, plastic, jik, cotton thread), Baby clothes, Other
- For Hammermill: Grinding services
- For Goat Rearing: Goats, manure

2h. Who is ***buying the products of the IGA***? *(Hint: what kinds of customers in terms of their characteristics, i.e. men, women, from far/near, jobs, etc)*
Probe for:

- For Agrodealership: Agro products (seed, fertilizer, chemicals), Birthing supplies (razor, plastic, jik, cotton thread), Baby clothes, Other
- For Hammermill: Grinding services
- For Goat Rearing: Goats, manure

2i. Pretend that ***I am a customer***. Be specific for each.

i. How do I make a purchase?
ii. How is the purchase recorded?
iii. How do I pay for the item(s)?
iv. How is this payment recorded (for money and in-kind)?
v. What do you do with that money or in-kind payment?

2j. Why would a ***customer come to this IGA*** and not a nearby business?
Probe for:

- Prices
- Stock variety
- Social enterprise (that the IGA benefits the MS)
- Staff friendliness

2k. Do **customers** know that the ***IGA supports the MWH***?

i. How do they know?
ii. What do they think of this connection?

<u>Theme 3: Financial Aspects of the IGA</u>

"We will now talk about the costs and profits of the IGA. "

3a. How do you decide to ***spend the money coming into the IGA***? Who is involved in making this decision?
Probe for:
- Routine costs of the IGA
- Savings

3b. Is the IGA making ***enough money to cover costs***?
i. Is it making extra on top of that?
ii. What do you do with the extra money?

3c. Are you able to ***save money***? Why or why not?
If yes, What is it saved for?

3d. How do you create and submit a ***report to the Governance Committee***?
i. What is in the report?
ii. How often are reports submitted?
iii. Who prepares it?
iv. Who reviews it?

3e. Please tell me about some of the ***good*** things about the IGA ***financial system***? Please provide specific examples. *(Hint: the IGA financial system is the books and reports that you fill our and the oversight by the Governance Committee)*

3f. Please tell me about some of the ***challenging*** things about the IGA ***financial system***? Please provide specific examples.

3g. What could be done to ***improve the financial system***?

<u>Theme 4: Linkages with the MWH</u>
"We will now talk about linkages between the IGA and the MWH. "

4a. Does the ***community in general know*** that the IGA helps support the MWH? *(If not answered previously)*
i. How do they know?
ii. What do they think about that?

4b. What kinds of things does the ***IGA do to support the MWH***?

i. Are there monthly payment to the MWH?
ii. How much is provided?
iii. Has the IGA missed any payments? Why?
iv. How do these payments occur?

4c. What is ***good*** about having an ***IGA linked with the MWH***? Tell me a story about a time when there was something good that came out of the IGA being linked with the MWH.

4d. What are some of the biggest ***challenges*** about being ***linked with the MWH***? Tell me a story of a time that was challenging because the IGA is linked to the MWH.
Probe for:

- Challenges at meetings with the governance committee treasurer or IGA sub-committee
- Ability to talk with governance committee treasurer or IGA sub-committee

Theme 5: Perspectives on Sustainability of the IGA

"We will now talk about the sustainability and future of the IGA. "

5a. What is your vision of the ***long-term sustainability*** of the IGA and the MS?
Probe for: Operational and Financial

5b. What do you think the ***biggest challenge will be to continuing the IGA*** after the implementing partner leaves?

5c. What do you think can be done to ***address these challenges***?

5d. What do you think the ***biggest challenge will be for supporting the MWH*** after the implementing partner leaves?

5e. What do you think can be done to ***address these challenges***?

We have completed this interview. Is there anything else you would like to tell me?

"Thank you for your time. Please feel free to reach out if you think of anything else that may be helpful for us to know regarding IGA."
