# Supplementary material for: Evaluating implementation effectiveness and sustainability of a maternity waiting homes intervention to improve access to safe delivery in rural Zambia: a mixed-methods protocol
Source: BMC Health Serv Res. 2020 Mar 12;20:191. doi: 10.1186/s12913-020-4989-x (PMC7068884; doi:10.1186/s12913-020-4989-x)
Supplement: Supplementary file 9 — Additional file 9. Focus Group Discussion Guide for Community Elders. [file 12913_2020_4989_MOESM9_ESM.pdf]

GROUP ID

Instrument ID: Form K4 ENGLISH

The MAHMAZ Project

Focus Group Discussion Guide with Community Elders

Target Audience:

1. *Mothers-in-Law and Elders in a study catchment area*
2. *>=15 years at last birthday, Elders >= 54 years*
3. *Has not previously participated in a FGD in the same year at another site or in another category*

Short screen:

Criteria	Response	Comments
SS1. Is this individual a community member or elder in a study catchment area?	Yes (1)<br>No (0)	If (0), do not proceed. Person is not eligible.
SS2. Is the individual >=15 years old?	Yes (1)<br>No (0)	If (0), do not proceed. Person is not eligible.
SS3. Has the individual previously participated in a focus group in the same year at the same or another site or other categories?	Yes (1)<br>No (0)	If (1), do not proceed. Person is not eligible.

Instructions for the interviewer

Step 1: Informed consent: Ask each participant for a few minutes of their time. Introduce yourself and the study. Begin the informed consent process as per the training.

Was informed consent obtained and documented for each participant before the start of the FGD?

YES ______ (proceed with roster and FGD)

NO ______ (STOP! Thank the participant for their time but ask any who did not agree to leave. Then proceed with the FGD.)

 GROUP ID

***Interviewer**: Read the following statement. Please repeat the statement translated into the local language based on primary languages used by the group.*

"Thank you for agreeing to participate in this interview. My name is _______________. I will be asking you the questions. My partner _______________ will be taking notes on the things you have to say. We will record this session.

We want to understand your views on maternity waiting homes (MWHs) and access to maternity care at the health facility in your community. Please feel free to tell us whatever you are comfortable sharing. As a reminder, please do not share anything you hear or said within this group outside of this group. You should also remember that you do not have to share anything you are not comfortable sharing. We will not link your information to your responses. There are no right or wrong answers, so please be honest and us what is true for you and your community. Are you ready to begin?"

Step 2: *Please complete the roster table for each participant as they sign in. Verify eligibility. This form will have an ID letter for each participant. Make sure that the note taker has the correct ID letter recorded on his/her notes prior to beginning and that participants have their correct "Letter Label".*

Step 3: *Proceed to the FGD. Start by reiterating the importance of confidentiality within the group. As you ask the questions, please probe to obtain as much information as possible for each question.*

Facilitator name ____________________ **Note-taker Name ____________________**

1. **FGD Date (DD/MM/YYY) ________________**
2. **Health Facility Name _________________**
3. **Health Facility ID _________________**
4. **District Name _________________**
5. **Province Name _________________**
6. **Time start (HH:MM) ________** **Time finish (HH:MM) ________**
7. **Supervisor initials __________**

INSTRUMENT K4:
Focus Group Discussion Guide for Community Elders

GROUP ID

Demographics: Community Members and Elders

**ID**	**Age at last birthday**	**Prev FGD this Round?**<br><br>**Yes (1)**<br>**No (0)**	**Eligibility confirmed?**<br>**Yes (1)**<br>**No (0)**<br><br>**If 1, proceed with the rest of the columns**<br><br>**If 0, stop and thank the participant for his/her time**	**Gender**<br><br>Male (1)<br>Female (2)	**Marital status:**<br><br>Married/ cohabiting (1)<br><br>Divorced (2)<br><br>Separated (3)<br><br>Widowed (4)<br><br>Never married (5)	**What is the highest grade you completed?**<br><br>If < grade 1, put 0.<br>If<br>> grade 12, put 13.	**How many live, biological children do you have?**	**# children, <18, yours or others, in house-hold**	**# adults >18, in household, including respondent**	**It is very worthwhile for someone like me/my wife/my daughter to stay in an MWH during the last weeks of a pregnancy***<br><br>*See below*	**Participant is a:**<br>(1) Mother-in-law<br>(2) Elder<br><br>*If both Elder and MIL, choose Elder*
**A**											
**B**											
**C**											
**D**											

*1 = Strongly agree, 2 = Agree, 3 = Neither agree nor disagree, 4 = Disagree, 5 = Strongly disagree

INSTRUMENT K4:
Focus Group Discussion Guide for Community Elders

GROUP ID

ID	Age at last birthday	Prev FGD this Round?<br><br>Yes (1)<br>No (0)	Eligibility confirmed?<br>Yes (1)<br>No (0)<br><br>If 1, proceed with the rest of the columns<br><br>If 0, stop and thank the participant for his/her time	Gender<br><br>Male (1)<br>Female (2)	Marital status:<br><br>Married/ cohabiting (1)<br><br>Divorced (2)<br><br>Separated (3)<br><br>Widowed (4)<br><br>Never married (5)	What is the highest grade you completed?<br><br>If < grade 1, put 0.<br>If<br>> grade 12, put 13.	How many live, biological children do you have?	# children, <18, yours or others, in house-hold	# adults >18, in household, including respondent	It is very worthwhile for someone like me/my wife/my daughter to stay in an MWH during the last weeks of a pregnancy*<br>*See below*	Participant is a:<br>(3) Mother-in-law<br><br>(4) Elder<br><br>*If both Elder and MIL, choose Elder*
**E**											
**F**											
**G**											
**H**											

Theme 1: Awareness, Utilization and Quality of the MWH

1a. Tell me what you know ***about MWHs in general***

i. What are they for?
ii. Who can stay there?

1b. ***Describe the state of the MWH*** in general at your health facility.

1c. What do people in your community say about the ***quality of the MWH*** at your health facility?

Probe for: (ask about each bullet separately, do not ask all at once!)

- Comfort (structure, furnishings, bedding, mosquito nets, cultural appropriateness)
- Safety (belongings safe, people staying there)
- Cleanliness
- Food availability or cooking space (structure, supplies)
- Classes offered there? (boredom, etc)
- Seasonal problems (leaking roofs, insect problems, etc)

1d. What do people in your community say about the ***management of the MWH*** at your health facility?

i. Who manages the MWH?
ii. How well is it managed?

1e. What do people in your community say about the ***linkages of the MWH with the*** health facility?
Probe for:

- Health facility staff presence at MWH
- Waiting women attending ANC visits at health facility

1f. What things make it ***hard for a woman to use the MWH***?
Probe for: (ask about each bullet separately, do not ask all at once!)

- Quality of MWH (safety, comfort, cultural appropriateness, other problems)
- Costs
- Food or cooking space
- Perceived quality of care at the health facility
- Family member opinions
- Other responsibilities at home
- Season

1g. What things make it ***easy for a woman to use the MWH***?
Probe for: (ask about each bullet separately, do not ask all at once!)

- Quality of MWH (safety, comfort, cultural appropriateness, other good things)
- Costs
- Food or cooking space
- Perceived quality of care at the health facility
- Family member opinions
- Other responsibilities at home
- Season

Theme 2: Community Ownership and Sustainability of MWHs

2a. How does the ***community contribute towards maintenance/upkeep*** of the MWH at your health facility?
Probe for:

- Cash contributions
- Food contributions
- In-kind labor (slashing, weeding, sweeping, etc)
- Maintenance
- Any other contributions?

2c. Can you think of other ways in which the community ***could contribute***?

2d. From your perspective, ***who owns the MWH***? Why?

Theme 3: Barriers and facilitators to facility delivery

3a. In your opinion, ***why do women who deliver at home*** do so instead of using a health facility?

Probe for issues related to: (ask about each bullet separately, do not ask all at once!)

- Distance/transport (ask for specifics)
- Costs (ask for specifics)
- Seasons (ask for specifics)
- Household responsibilities
- Permission or support from family members
- Perceptions of health facility and quality of care
- Perceptions of pregnant women's or child's health risk
- Myths and misconceptions about home or facility delivery

3b. Are there penalties for delivering at home?
If yes:

i. What are those penalties?
ii. Who enforces these penalties?

3c. In your opinion, does a MWH ***influence a woman/family's decision*** to deliver at a facility? Why or why not?

3d. Why might a pregnant woman in your community who is high-risk or who lives far from the facility ***choose not to stay*** in the MWH before delivery?

Theme 4: Preparedness and Costs

4a. How do women in your community ***plan for delivery***?

Probe for issues related to: (ask about each bullet separately, do not ask all at once!)

- Birth supplies including plastic sheets, gloves, sterile blade, etc
- Baby clothes
- Travel
- Food
- Accommodation
- Payments for delivery, informal payments, in-kind donations)

4b. About ***how far along*** in a pregnancy do women begin planning for delivery?

4c. Do women ***move from their homes*** to their mother's/relative's homes in preparation for delivery?
If yes:
(i) Does this happen for each pregnancy?
(ii) At what point in their pregnancy do women move?
(iii) Why do women move to their mother's home?
(iv) Where do women deliver from when they move to their mother's home?
(v) If they deliver at a facility, is it the facility closest to their mothers' home or elsewhere?

4d. If a woman is delivering at ***home, what costs*** will she have (monetary & in-kind)? Be specific.
Probe for:
- Birth supplies including plastic sheets, gloves, sterile blade, etc
- Baby clothes
- Travel
- Food
- Payments for delivery, informal payments, in-kind contributions to birth attendant

4e. If a woman is delivering at a ***facility or hospital***, how would the ***costs*** be different from if she were delivering at home?
Probe for:
- Birth supplies including plastic sheets, gloves, sterile blade, etc
- Baby clothes
- Travel
- Food
- Accommodation
- Payments for delivery, informal payments, in-kind contributions to birth attendant

4f. How much do women normally ***spend on birth supplies***? Where do women purchased them?
Probe for:

- Plastic sheet
- Gloves
- Sterile blade/razor
- Clamps
- Anything else?

4g. Where do women normally ***purchase baby clothes***?

4i. What ***modes of transport*** do women use to get to the health facility for delivery?

4k. How much do women normally ***spend on food*** for during their stay at the MWH? (Separate from what they share with home)

4l. When women plan for delivery ***do they save money***?
If yes:

i. How do they save money?
i. Do they normally save enough?

If no, why not?

4m. What are the main ***challenges associated with saving money*** for delivery?
Probe with:

- Competing needs (accidents, illness, basic needs)
- Safety of storing money

4n. Do other people in the ***community contribute*** (money or in-kind) to a woman's delivery savings?
If yes:

i. What do they contribute? (money or in-kind)
ii. Who contributes?
iii. Who manages the contributions?

4o. Are there any ***savings groups*** in your communities?

If yes: Do women use these groups to save money in preparing for delivery?

Theme 5: Tailoring

5a. Are you aware of ***anyone making baby clothes, reusable sanitary pads, or other items*** in the catchment area or nearby? Please explain

If yes:

i. What items are made?
ii. In your opinion, how is the ***quality***?
iii. How is the ***cost***?
iv. How is the ***availability*** of the tailoring clothes?

This is the end of the Focus Group. Please thank the participants for their time.
